# A Rare Case of Bullous Pemphigoid Triggered by Overlapping Immune Dysregulation From HIV and Hodgkin Lymphoma: A Case Report

**DOI:** 10.7759/cureus.95852

**Published:** 2025-10-31

**Authors:** Ismael Palacio, Kristal Arraut, Kaira Portalatin, Amir A Estil-las, Martena Grace

**Affiliations:** 1 Internal Medicine, Larkin Community Hospital, Miami, USA; 2 Medical School, University of Medicine and Health Sciences, Basseterre, KNA; 3 Medical School, Ross University School of Medicine, Miramar, USA

**Keywords:** autoimmune, blistering skin disease, bullous pemphigoid, hiv/aids, hodgkin lymphoma, immune regulation, tense bullae

## Abstract

Bullous pemphigoid (BP) is the most common autoimmune subepidermal blistering disorder, typically affecting elderly patients. Most cases are idiopathic, but BP has been reported to occur as a paraneoplastic phenomenon or in association with immune system abnormalities. We present a unique case of biopsy-confirmed BP in a 43-year-old man with advanced HIV infection (CD4 count: 4 cells/µL) and untreated classic Hodgkin lymphoma. The patient developed progressive tense bullae and desquamating skin lesions involving approximately 15% total body surface area, requiring debridement and subsequent transfer to a burn center for wound care. Laboratory studies demonstrated severe immunosuppression with a CD4 count of 4 cells/µL and pancytopenia. Histopathologic examination revealed subepidermal blistering with an eosinophil-rich infiltrate, and direct immunofluorescence showed linear deposition of IgG and C3 along the dermoepidermal junction. Serologic testing confirmed the presence of BP180 and BP230 autoantibodies. Management was complicated by the patient’s immunosuppression, limiting the use of systemic corticosteroids and delaying wound healing. This case highlights an uncommon clinical intersection of BP, advanced HIV, and Hodgkin lymphoma.

## Introduction

Bullous pemphigoid (BP) is the most common autoimmune subepidermal blistering disorder, commonly affecting patients between the ages of 60 and 80 [1]. The estimated annual incidence is approximately six to 13 new cases per million people in the United States [1]. Clinically, BP must be differentiated from other blistering conditions such as pemphigus vulgaris, Stevens-Johnson syndrome/toxic epidermal necrolysis, and bullous drug eruptions, which can present with similar overlapping skin lesions. BP is characterized by the development of tense bullae and erythematous, pruritic plaques that typically spare the mucous membranes [2]. The disease is pathologically defined by autoantibodies targeted against BP180 and BP230, which are two structural hemidesmosomal proteins essential for dermoepidermal adhesion. These autoantibodies trigger complement activation and subsequent subepidermal blister formation [3]. 

While the majority of BP cases are idiopathic, secondary forms can occur in association with medications, infections, radiation, or malignancies [1]. Paraneoplastic BP is rare but has been reported most frequently in connection with hematologic malignancies, including Hodgkin and non-Hodgkin lymphomas [4-7]. These associations suggest that neoplastic processes may act as immune triggers in genetically or immunologically susceptible individuals.

The occurrence of BP in patients with HIV is particularly uncommon, with only a few case series and reports published [8-10]. The coexistence of BP in the setting of both HIV and Hodgkin lymphoma is exceedingly rare, representing a convergence of profound immunosuppression and paraneoplastic immune dysregulation. Immunosuppression typically results in decreased autoantibody production; however, aberrant immune reconstitution or concurrent malignancy may contribute to the development of autoimmune blistering disease in this population.

The objective of this case is to describe the clinical and pathologic confirmation of BP in a patient with advanced HIV and untreated Hodgkin lymphoma, and to discuss potential mechanistic links and management challenges in this unique setting. This case illustrates an unusual clinical intersection that posed diagnostic and therapeutic challenges and underscores the need to consider autoimmune blistering disorders in immunocompromised patients.

## Case presentation

**Figure 1:**
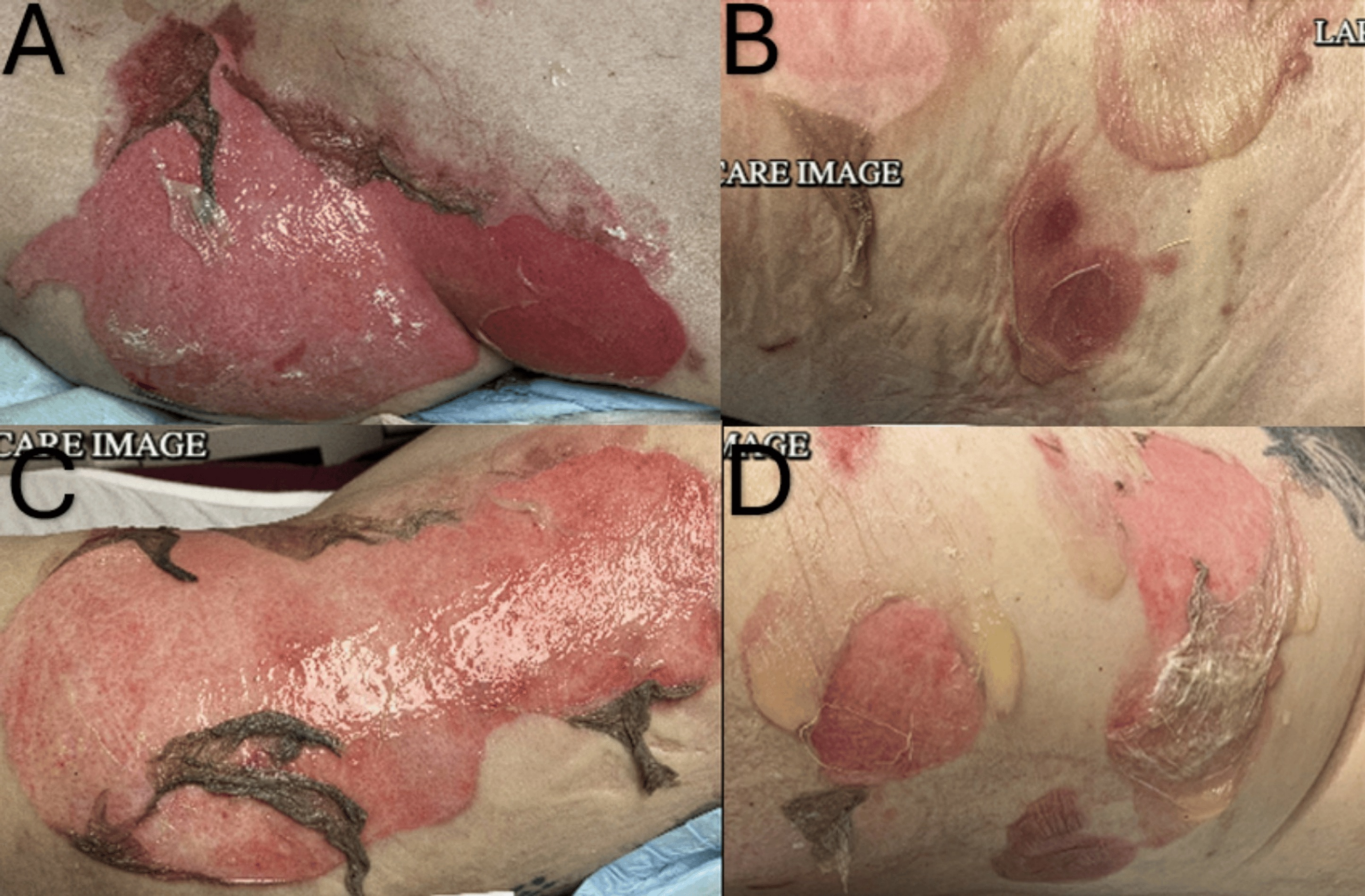
Patient skin lesions The image demonstrates four panels (A, B, C, D) of the patient's skin lesions. A: image of the left hip and thorax, B: image of the right lateral flank, C: image of the left lateral flank, D: image of the right lateral thorax.

We present a 43-year-old male with a past medical history of HIV diagnosed seven months prior, untreated classic Hodgkin lymphoma, nodular sclerosis type, diagnosed two months before presentation, who presented from a correctional facility on February 7, 2025, with progressive blistering and sloughing of the skin. The patient had been prescribed bictegravir/emtricitabine/tenofovir (Biktarvy) for HIV management, but reported inconsistent adherence, with uncertain timing of his most recent doses prior to admission. He had also been prescribed trimethoprim-sulfamethoxazole (TMP-SMX) and azithromycin for opportunistic infection prophylaxis; however, adherence to these medications was unclear. Notably, according to both patient reports and medical records, the cutaneous lesions began before any recent medication changes or re-initiation, making drug-related bullous eruption or Stevens-Johnson syndrome/toxic epidermal necrolysis less likely. The patient reported a three-week history of bullous eruptions that initially appeared over the left groin and hip and progressively spread to the abdomen, thorax, and bilateral extremities (Figures 1A-1D), notably sparing the oral mucosa. The lesions were painful, rated at a 7/10 in intensity, erythematous and edematous, with a negative Nikolsky and Asboe-Hansen sign, and intensely pruritic. He denied weight loss, night sweats, or bleeding, although he arrived febrile to 101.2°F upon admission. Subsequent blood cultures were negative. 

**Figure 2:**
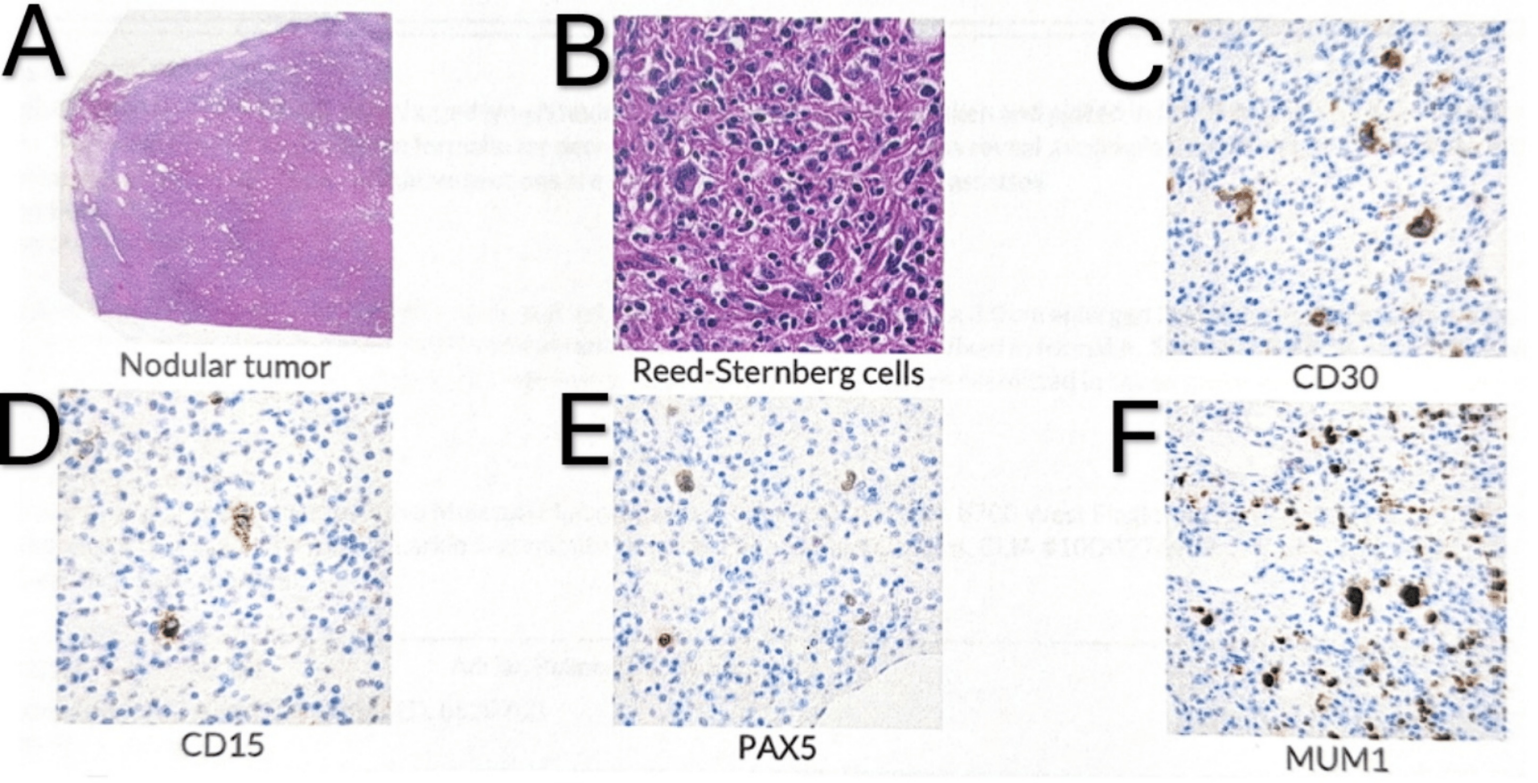
Microscopic findings of the excisional lymph node biopsy with corresponding immunohistochemical stains. A: Microscopy shows a nodular tumor architecture with broad fibrous bands compartmentalizing the lesion. B: Higher-power hematoxylin and eosin (H&E) section demonstrates numerous characteristic Reed-Sternberg cells scattered throughout an inflammatory background. C: Immunohistochemistry for CD30 shows strong membranous and perinuclear Golgi-type positivity in large atypical cells. D: CD15 highlights scattered large cells with weak cytoplasmic staining. E: PAX5 demonstrates weak and heterogeneous nuclear expression in tumor cells. F: MUM1 shows nuclear positivity in neoplastic cells.

Two months prior, the patient underwent a left inguinal lymph node excisional biopsy (Figure 2), confirming classic Hodgkin lymphoma (nodular sclerosis, EBV-negative). He was lost to follow-up and never initiated systematic therapy. HIV had been managed with Biktarvy, but medication adherence remained inconsistent. He had previously been prescribed TMP-SMX and azithromycin for opportunistic infection prophylaxis, although recent compliance was uncertain. 

On examination, multiple tense bullae and partial-thickness desquamating lesions with serous exudate were present over the left hip, trunk, inguinal regions, and extremities. Newer vesicular lesions continued to form, while older lesions were in the healing phase. There was no mucosal involvement. Bilateral pitting edema of the lower extremities was noted. Laboratory workup on admission revealed pancytopenia (Table 1). CT imaging demonstrated splenomegaly, diffuse intra-abdominal lymphadenopathy, a 6 mm pulmonary nodule, and a 2 x 5 x 2 cm left groin fluid collection at the prior lymph node biopsy site. 

**Table 1:** Admission labs Laboratory values on admission demonstrating pancytopenia. Abnormal values are bolded.

Admission labs	Values	Units	Reference interval
White blood cell (WBC)	1.8	x10^3^/µL	3.4-10.8
Red blood cell (RBC)	2.83	x10^6^/µL	3.77-5.28
Hemoglobin (HGB)	7.5	g/dL	11.1-15.9
Hematocrit (HCT)	23.9	%	34.0-46.6
Mean corpuscular volume (MCV)	84.5	fL	79-97
Mean corpuscular hemoglobin (MCH)	26.5	pg	26.6-33.0
Mean corpuscular hemoglobin concentration (MCHC)	31.4	g/dL	31.5-35.7
Red cell distribution width (RDW)	24.1	%	11.7-15.4
Platelet (PLT)	132	x10^3^/µL	150-450
Mean platelet volume (MPV)	10.80%	%	Not established
%NEUT	78.80%	%	Not established
%LYMPH	6.70%	%	Not established
%MONO	10.00%	%	Not established
%EOS	0.60%	%	Not established
%BASO	0.00%	%	Not established
#NEUT	1.42	x10^3^/µL	1.8-8.0
#LYMPH	0.12	x10^3^/µL	0.7-3.1
#MONO	0.18	x10^3^/µL	0.1-0.9
#EOS	0.01	x10^3^/µL	0.0-0.4
#BASO	0	x10^3^/µL	0.0-0.2
Erythrocyte sedimentation rate (ESR)	60	mm/h	<15 mm/h

Dermatology and plastic surgery were consulted. Empiric broad-spectrum antibiotics, cefepime and TMP-SMX, and wound care with silver sulfadiazine were initiated. Corticosteroid therapy was deferred due to immunosuppression. Given the patient's extensive open lesions and high infection risk, steroids were withheld during hospitalization, with plans for outpatient reassessment once immune recovery and skin healing allowed. Punch biopsies of peri-lesional skin (left medial thigh 2/9/25; left groin, 2/10/25) revealed subepidermal blister formation with an eosinophil-rich dermal infiltrate on hematoxylin and eosin staining. Direct immunofluorescence demonstrated linear deposition of IgG and complement component C3 along the dermoepidermal junction. Serum BP180 and BP230 autoantibodies resulted in 11 U/mL (positive >9 U/mL) and 37 U/mL (positive >9 U/mL), thus indicating a diagnosis of BP. On hospital day five, the patient underwent debridement of superficial partial-thickness bullous lesions involving approximately 15% total body surface area, followed by skin substitute placement. Postoperatively, nutritional support, strict fluid and electrolyte monitoring, and reverse isolation precautions were maintained. 

The patient remained hemodynamically stable and afebrile. He was subsequently transferred to a regional burn center for advanced wound care. Outpatient follow-up was arranged with dermatology for biopsy and autoantibody results, hematology-oncology for lymphoma staging and treatment initiation, and infectious disease for optimization of antiretroviral therapy and opportunistic infection prophylaxis.

## Discussion

BP is an autoimmune subepidermal blistering disease usually affecting elderly individuals and is rarely encountered in younger or immunocompromised populations. This case presents a unique presentation of BP in a patient with HIV infection and untreated Hodgkin lymphoma, highlighting an unusual potential synergy of pathologies that likely contributed to disease development. Reports of BP occurring in the setting of either hematological malignancy or HIV infection exist, but cases involving both are rare. In lymphoma-associated BP, tumor-associated antigens or cytokine dysregulation may aberrantly activate B cells and potentially induce the formation of autoantibodies against hemidesmosomal antigens (BP180 and BP230). In addition, poor adherence to antiretroviral therapy could have resulted in intermittent immune activation, potentially amplifying autoimmune activation through mechanisms like those in immune reconstitution inflammatory syndrome (IRIS). To clarify the temporal relationship between these factors, a concise clinical timeline (Table 2) has been included.

**Table 2:** Clinical timeline of disease progression and management This table is a visual summary of the temporal relationship between key clinical events in the patient's course, including HIV diagnosis, Hodgkin lymphoma biopsy, onset of skin lesions, hospital presentation, skin biopsies, surgical debridement, and transfer to burn center.

Event	Date
HIV diagnosis	July 2024
Lymph node biopsy (Hodgkin lymphoma)	December 2024
Onset of bullous skin lesions	January 2025
Hospital presentation	February 7, 2025
Punch biopsies (left medial thigh, left groin)	February 10, 2025
Surgical debridement (15% TBSA)	February 12, 2025
Transfer to burn center	February 15, 2025

BP arises from autoantibodies directed against the hemidesmosomal proteins BP180 and BP230, leading to complement activation, inflammatory cell infiltration, and dermal-epidermal separation [11]. The precise mechanism by which malignancy may trigger BP is not fully understood, but popular hypotheses include altered antigen presentation, molecular mimicry, tumor-induced immune dysregulation, and epitope spreading [12]. Current research has confirmed that the pathogenesis of BP is largely attributed to the binding of autoantibodies to the NC16A domain of BP180. This initial event is what recruits the classical pathway of the complement cascade, in turn recruiting neutrophils, eosinophils, mast cells, and release of proteases and reactive oxygen species that ultimately damage and destroy structural components at the dermal-epidermal junction [12,13]. Furthermore, the binding of autoantibodies to BP180 may induce internalization or total depletion of BP180 from the cell surface, weakening the dermal-epidermal junction, independent of complement recruitment [11].

BP230 auto-antibodies are also associated with the pathogenesis of BP; however, their role in BP is unclear. Some experimental models suggest that anti-BP230 IgG alone may generate blistering under some circumstances [14].

The presence of an advanced HIV infection and untreated Hodgkin lymphoma in our patient adds complexity to the interpretation of disease pathogenesis. Autoimmune blistering disorders are rare in advanced HIV, most likely due to depleted or low levels of circulating CD4+ helper T cells being unable to stimulate autoantibody production. Nonetheless, HIV is also associated with chronic immune activation and polyclonal B-cell stimulation, which may predispose individuals to autoimmunity under certain triggers. In our case, the underlying lymphoma may have provided a stimulus for immune dysregulation in a background of HIV, precipitating BP. 

The therapeutic approach in this patient was complicated by the severe immunosuppression. Following transfer to the burn center, the patient demonstrated gradual re-epithelialization of the affected areas with no new bullae formation. Antiretroviral therapy was re-initiated, and hematology-oncology planned to begin systemic therapy for Hodgkin lymphoma once immune status improved. One of the key therapeutic options for BP is the use of glucocorticoids or systemic steroids, which were avoided due to the immunosuppression [15]. Instead, management thus emphasized wound care, prevention and treatment of infection, nutritional support, and surgical debridement rather than conventional immunosuppression. This case demonstrates the nuance of balancing disease control with the risks posed by an immunocompromised patient. 

Our case demonstrates the possibility of BP as a differential diagnosis even in severely immunocompromised individuals, considering underlying malignancy as a possible trigger. Further studies are warranted to determine how combined immune abnormalities: HIV and lymphoma, may trigger autoimmune blistering diseases and to guide safe therapeutic strategies.

## Conclusions

This case illustrates a rare presentation of BP arising in the dual presence of advanced HIV and untreated Hodgkin lymphoma. The coexistence of immunosuppression and malignancy likely contributed to the development of BP through overlapping mechanisms of immune dysregulation. Accurate diagnosis required careful clinicopathologic correlation, and management demanded a tailored approach that balanced disease control with the risks of more profound immunosuppression. Clinicians should maintain a high index of suspicion for BP in atypical hosts and recognize that its occurrence may be associated with, rather than directly caused by, underlying malignancy. Continued reporting of such rare presentations is necessary to improve understanding of disease mechanisms and to guide management strategies in complex immunologic settings.
